# Insights on Designing the MINT (Micro-Interventions for Navigating Tension) Chatbot System for Stress Management: Collaborative Autoethnography

**DOI:** 10.2196/89876

**Published:** 2026-10-27

**Authors:** Marco Bolpagni, Sofia Asteria Piffer, Marco Pozza, Munesu Maminimini, Silvia Gabrielli

**Affiliations:** 1 Department of General Psychology, University of Padua, Via Venezia, 8, Padua, Veneto, 35131, Italy, (+39) 049 827 6485; 2 Centre for Digital Health and Wellbeing, Digital Health Research, Fondazione Bruno Kessler, Trento, Italy

**Keywords:** digital health, stress management, digital microinterventions, mental health chatbots, ecological momentary assessment, just-in-time adaptive interventions, collaborative autoethnography, user engagement, wearable sensors, generative AI

## Abstract

**Background:**

Digital microinterventions are increasingly used to support stress self-management in everyday life. While previous research has examined their feasibility, adherence, and preliminary effectiveness, much less is known about how these interventions are experienced over extended periods of real-world use.

**Objective:**

This study used collaborative autoethnography (CAE) to examine the everyday experience of living with MINT (Micro-Interventions for Navigating Tension), a Telegram-based chatbot for stress management. Our goal was not to evaluate clinical outcomes but to understand how scheduled microinterventions fit into everyday life, when they were ignored, and when people relied on other ways of coping instead, and to identify implications for the design of future digital mental health tools.

**Methods:**

We conducted a 4-week CAE involving 5 digital mental health researchers, who used the MINT chatbot as both participants and researchers. The experience was documented through multiple qualitative and quantitative sources, including reflective diaries, ecological momentary assessments (EMAs), workshop discussions, and wearable data, and analyzed collaboratively using reflexive thematic analysis (RTA).

**Results:**

Six interconnected themes described how MINT was experienced in everyday life: (T1) the importance of timing and emotional receptivity, (T2) the need for reciprocity and relational continuity, (T3) the tendency to favor existing coping strategies over suggested exercises, (T4) the meaning of nonengagement during periods of high stress, (T5) the collapse of self-initiated use after reminders were removed, and (T6) how everyday experiences with consumer wearables and general-purpose large language models reshaped expectations of digital mental health systems.

**Conclusions:**

Our findings show that CAE complements conventional evaluations of digital mental health interventions by revealing aspects of everyday use that are difficult to capture through feasibility studies, large-scale trials, or looking at usage data. Beyond the specific case of MINT, they suggest that future stress-management systems should better account for users’ receptivity, provide greater continuity across interactions, build on existing coping strategies, and help users move from externally prompted engagement to autonomous self-management.

## Introduction

Stress is a pervasive challenge that affects psychological well-being, cognitive functioning, and overall quality of life [1]. Despite its prevalence, individuals often lack accessible, personalized tools to help them recognize and regulate stress. Digital mental health interventions (DMHIs), including mindfulness apps [2], chatbots [3], ecological momentary interventions (EMIs) [4], and wearable devices [5], have attempted to fill this gap by delivering support outside clinical environments. One approach that has attracted growing attention is the use of digital microinterventions. Microinterventions are short, structured therapeutic activities, such as cognitive reappraisal [6], guided breathing [7], positive imagery [8], or mindfulness exercises, that can be completed in under a few minutes [9]. Their brevity and adaptability make them well suited for real-world stress self-management, where users often have only small windows of time available. Research has already explored several ways of delivering microinterventions in everyday settings. For example, Microsoft researchers [10] have developed a just-in-time adaptive intervention (JITAI) system for workplace stress management, showing that context-triggered support, derived from indicators, such as email load, meeting frequency, time of day, facial expression cues, and heart rate, can be feasibly delivered and meaningfully integrated into daily activities. Other work [11] has focused on wearable-based approaches, where smartwatches periodically prompt users to report how they feel and then offer brief, breathing-oriented microinterventions to support emotional regulation during moments of elevated arousal. Further analysis [12] has evaluated a range of cognitive behavioral therapy (CBT)–based techniques to identify particularly promising exercises and examine how different stress levels may align with specific intervention strategies. Finally, a study [13] conducted a microrandomized trial with parents using the commercial mindfulness app Headspace to assess the feasibility and acceptability of delivering mindfulness-based microinterventions for stress regulation in daily life.

However, most field evaluations focus on feasibility, adherence, or preliminary outcomes and not on the lived experience of interacting with microinterventions across an extended period. As a result, while many of the practical challenges surrounding engagement with digital microinterventions have already been identified, we still know comparatively little about how these challenges play out in everyday life: how such interventions fit (or fail to fit) into daily routines, how users make sense of the activities, how emotional context shapes engagement, or how motivation fluctuates across weeks. This limitation reflects a broader concern in digital mental health research. Large trials and log-based analyses, while valuable for quantifying use, often miss how engagement is shaped by everyday contexts and circumstances, making it difficult to explain why people sustain or abandon DMHIs. Prior work [14] on “trial bias” further suggests that engagement observed in controlled studies may not reflect real-world use, underscoring the need for methodologies that foreground real-life contexts and subjective experiences. To better understand these everyday dynamics, researchers have increasingly turned to methods that foreground lived experience. This shift has prompted researchers both to involve users as collaborators, helping to interpret how digital mental health tools shape and are shaped by identity, emotion, and daily routines [15,16], and to turn the analytic lens inward, using autoethnographic methods to generate richly contextual insights from their own experiences [17]. This growing interest is reflected in recent work. Authors of the study [18], for example, used autoethnography to explore how low-tech event-logging can be used for smartphone disengagement. Another study [19] tracked 16 weeks of sleeping with a companion robot, highlighting how emotional and relational dynamics emerge through extended use. More recently, the authors of the study [20] used a duoethnographic design to examine how wearable-triggered prompts and large language model (LLM)–based interventions unfold in daily life.

This paper presents a collaborative autoethnography (CAE) [21] of MINT (Micro-Interventions for Navigating Tension), a Telegram chatbot delivering brief stress-management exercises through scheduled prompts and user-initiated access. Our goal was not to evaluate clinical efficacy or stress-contingent delivery. Instead, we used MINT as a formative case to examine a more basic design problem: how scheduled microinterventions are taken up, resisted, or displaced in everyday life. Over 4 weeks, 5 researchers used the system while documenting their own experiences as both users and designers. Through this case, we explore the conditions under which scheduled microinterventions fail to become part of a person’s own coping repertoire and derive implications for the design of future stress-management systems.

## Methods

### Overview of the MINT Prototype

MINT is a Telegram chatbot designed to support stress self-management through brief, evidence-based microinterventions. The system aims to provide immediate stress relief while helping users develop skills for everyday resilience by teaching exercises that foster resilience. Users can log mood, stress, sleep, physical activity, and coping strategies, as well as record reflections in a diary-style format (Figure 1). Exercises (microinterventions) are available both through scheduled (“push”) delivery and user-initiated (“pull”) access, allowing users to actively select both the exercise and its delivery style. MINT’s interventions are grounded in CBT techniques and include cognitive reappraisal [6], controlled breathing [7], positive mood induction [8], smiling [22], and guided visualization of one’s best possible self [23]. Each exercise lasts under 5 minutes and can be tailored to match the user’s preferred learning style according to the visual, aural, read/write, kinesthetic (VARK) framework [24], adapting its delivery as a visual diagram, audio guide, written instructions, or kinesthetic prompt (see an example in Multimedia Appendix 1). VARK variants were intentionally introduced to support learning and skill acquisition and to eventually help users internalize stress-management techniques. The flows of exercises were designed by the research team and delivered through scripted conversations in the chatbot interface.

**Figure 1.**
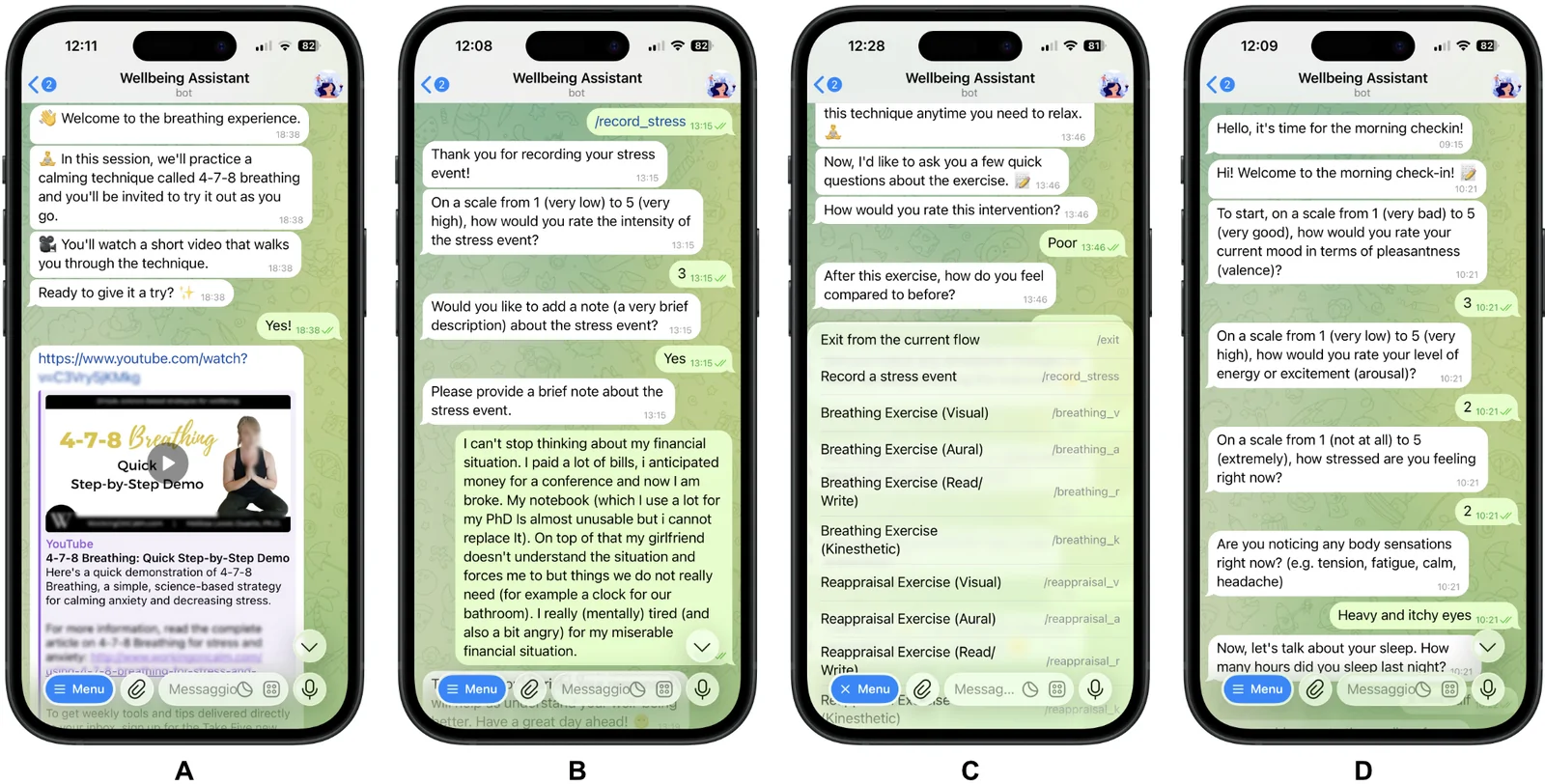
Screenshots of the MINT (Micro-Interventions for Navigating Tension) Telegram chatbot illustrating core interaction types: (A) delivery of a scheduled microintervention, (B) manual stress and mood recording, (C) menu for user-initiated (“pull”) selection of any exercise, and (D) ecological momentary assessment (EMA). All information related to real people has been censored. All remaining conversations are fictional; therefore, consent is not required.

At the start, MINT collects an initial user profile, including personality (Big Five Inventory-10 [BFI-10; 25]), coping style (COPE-28 [Coping Orientation to Problems Experienced-28; 26]), cognitive style (Rational Experiential Inventory-13 [REIm-13; 27]), and learning style preferences (VARK v8.01 [28]); while most measures are used for research purposes, only the VARK preference guides personalization. Optional wearable integration is also supported and enables passive monitoring of physiological signals, such as electrodermal activity (EDA), heart rate variability (HRV), and skin temperature, to gather exploratory insights into the feasibility of wearing a smartwatch for multiple weeks and user perceptions of such data. Architecturally, MINT was developed as a rule-based conversational agent using Node.js and the grammY framework for Telegram API integration. Interventions and ecological momentary assessment (EMA) delivery followed scripted, finite-state decision trees, with user responses, logged metrics, and interaction timestamps stored in a MongoDB database. Scheduled push notifications were triggered via automated background cron jobs on a dedicated self-hosted server.

### Study Design

As anticipated in the *Introduction*, we chose CAE to explore our lived, day-to-day experiences of using the MINT chatbot. The CAE [21] is a qualitative methodology in which researchers document and interpret their own experiences in a systematic and collective way, making visible aspects of everyday experience that are difficult to capture otherwise.

### Participants and Researcher-Participant Positionality

All 5 authors served as researchers-participants (RPs; Table 1), engaging with MINT over a 4-week period in June 2025 while reflecting on its role in our daily lives. Each of us had prior experience studying or designing digital mental health technologies, and 1 (RP2) had led the development of the MINT prototype. Recognizing that this shared expertise inevitably shaped our expectations and interpretations, we paid explicit attention to positionality throughout the study, regularly discussing in debriefing sessions how our disciplinary backgrounds, design knowledge, and personal histories influenced our experiences and sensemaking of MINT.

**Table 1.** Characteristics of the 5 researchers-participants (RPs), including their roles, backgrounds, involvement in MINT (Micro-Interventions for Navigating Tension) development, PSS-10^a^ scores, and visual, aural, read/write, kinesthetic (VARK) preferences.

ID	Sex/age (y)	Role (background)	Expertise	Involvement in MINT	PSS-10 (T0→T1)^b^	VARK (1st→2nd)
RP1	Female/55	Senior researcher (PhD in psychology, MSc in psychology)	HCI^c^ digital health (psychology interventions)	Supervisor: aware of the overall concept	17→14	K^d^→R
RP2	Male/33	PhD researcher (MSc in DS^e^, BSc in psychology)	Digital health (AI and psychology interventions)	Lead developer: built the bot and refined exercises/EMA^f^ logic	17→17	K→V^g^
RP3	Male/30	PhD researcher (MSc in CS^h^, BSc in CS)	Digital health (AI)	Regular research group member: user only	23→22	K→R^i^
RP4	Female/25	Technologist (MA in UX^j^ psychology, BSc in design)	UX psychology	Designer: found and drafted many of the exercises	18→11	K→V
RP5	Male/25	PhD researcher (MSc in CS, BEng in electronics)	Digital health (engagement)	Regular research group member: user only	23→23	K→V

a PSS-10: Perceived Stress Scale.

b T0, measurement at the beginning, and T1, measurement at the end of the autoethnography.

c HCI: human computer interaction.

d K: kinesthetic.

e DS: data science.

f EMA: Ecological Momentary Assessment.

g V: visual.

h CS: computer science.

i R: read/write.

j UX: user experience.

### Procedures

We interacted with MINT through the Telegram bot for 28 consecutive days following a structured weekly progression (Table 2), with each week focusing on different modes of engagement and intervention delivery to introduce frictions, sustain learning, and elicit responses under different conditions.

**Table 2.** Overview of the 4 experimental phases (weeks 1‐4) and their respective intervention delivery strategies.

Week	Phase name	Description	Key focus/purpose
#1	Preferred modality delivery	One daily afternoon microintervention delivered in the participant’s dominant VARK^a^ learning preference.	Establish a baseline of “comfortable,” intuitive, and preferred engagement.
#2	Nonpreferred modality delivery	Daily interventions continued but were intentionally mismatched with the participant’s VARK preferences.	Create mild friction; explore resistance, adaptability, and emotional reactions to nonpreferred formats.
#3	Participant-selected activities	No specific exercises suggested; participants received only 1 daily prompt inviting them to choose any intervention themselves.	Shift responsibility to the user; emphasize autonomy and the cognitive/emotional effort of self-selection.
#4	Free-use period	All scheduled prompts removed; participants could interact (or not interact) with MINT^b^ entirely on their own terms.	Capture fully self-directed patterns of engagement and disengagement.

a VARK: visual, aural, read/write, kinesthetic.

b MINT: Micro-Interventions for Navigating Tension.

Each weekday, we received an EMA in the morning at 9:15 AM, another EMA in the afternoon at 7:15 PM, and a microintervention at 6:15 PM (Table 2). To reduce burden, we received no interventions on weekends, and on these days we only completed the morning and afternoon EMAs. At the end of the 4 weeks, we hosted 2 in-person postintervention reflective workshops [29] (each approximately 90 min long) to revisit our experiences and develop a shared interpretation of the 4-week journey. The sessions were audio-recorded and transcribed to support subsequent analysis.

### Ethical Considerations

This study was conducted in full accordance with the Declaration of Helsinki and with current Italian regulations governing research involving human participants. In this case, in line with Italian Law No. 3/2018 [30] (Art. 2, paragraphs 7 and 10), formal ethics committee approval was not required because the investigation focused exclusively on the usability and lived experience of a prototype digital intervention, did not involve patients or vulnerable individuals, and did not entail any clinical treatment. Nevertheless, all RPs provided voluntary, written informed consent before enrollment, were free to withdraw from the study at any time without consequence, and were fully informed about data handling procedures.

### Data Collection

To capture our experiences from different perspectives, we collected multiple forms of data throughout the 4-week study period. Each of us kept a personal diary, noting episodes of stress, how the chatbot fit into our daily routines, and our emotional reactions to each interaction (eg, comfort, resistance, irritation, or relief). These diaries served as our primary narrative record that captured not only what happened but also how it felt to navigate ordinary routines with the system in the background. Alongside these individual accounts, we maintained a shared reflexive log where we posted brief observations, patterns we noticed, or questions we wanted to revisit later. These shared notes often prompted new reflections in our personal diaries, creating a fluid back-and-forth between private and communal sense-making. The interactions with MINT also generated an additional source of data. Our EMA responses and free-text messages, collected through the chatbot, provided brief snapshots of mood and stress that enriched the corpus of personal reflections. Finally, 3 of us used wearable devices for continuous physiological monitoring: RP2 wore an EmbracePlus (Empatica Inc) [31], RP4 wore a Galaxy Watch 4 (Samsung Electronics) [32], and RP3 wore a Watch SE2 (Apple Inc) [33]. These devices generated continuous physiological data throughout the study, complementing our subjective accounts while also revealing what it was like to live with continuous stress monitoring.

### Data Analysis

All qualitative data were analyzed through reflexive thematic analysis (RTA) [34], following the 6-phase analytic process described by Braun and Clarke [35]. Consistent with the logic of CAE, the 2 postintervention workshops served as the primary site of analysis, providing a space for collective sense-making through which researchers reflected on, interpreted, and negotiated the meaning of their experiences. These discussions served as the main point of theme generation, capturing shared and divergent experiences with the MINT chatbot. The resulting themes were then revisited across the broader dataset, including personal diaries, the shared reflexive log, stress entries, EMAs, and summaries of wearable data, to refine and contextualize the emerging interpretations. All materials were stored in a shared Google Drive folder, cleaned for clarity, and anonymized using nonidentifying placeholders (eg, [NAME] to replace actual names of people cited in the notes/transcripts) and participant codes (RP1-RP5). Throughout the analysis, we documented emerging themes and analytic decisions in a shared codebook. The final themes reported in this paper reflect what emerged through this collaborative process of integration, shared discussions, and individual reflections across all data sources.

## Results

### Themes Identified Through Interactions With MINT

Over the 4 weeks, our collective engagement with MINT evolved in ways that were often surprising and sometimes frustrating. Across the entire corpus analyzed, 1 pattern became clear: our responses to the chatbot were not simply reactions to individual exercises. They depended just as much on when the prompts arrived, what we were already doing to cope, how much effort the interaction required, and whether the system seemed able to give anything back. We identified 6 interrelated themes (Figure 2) that describe core design challenges that emerged through interaction with MINT (more details on themes, subthemes, and coding instances are reported in Multimedia Appendix 2).

**Figure 2.**
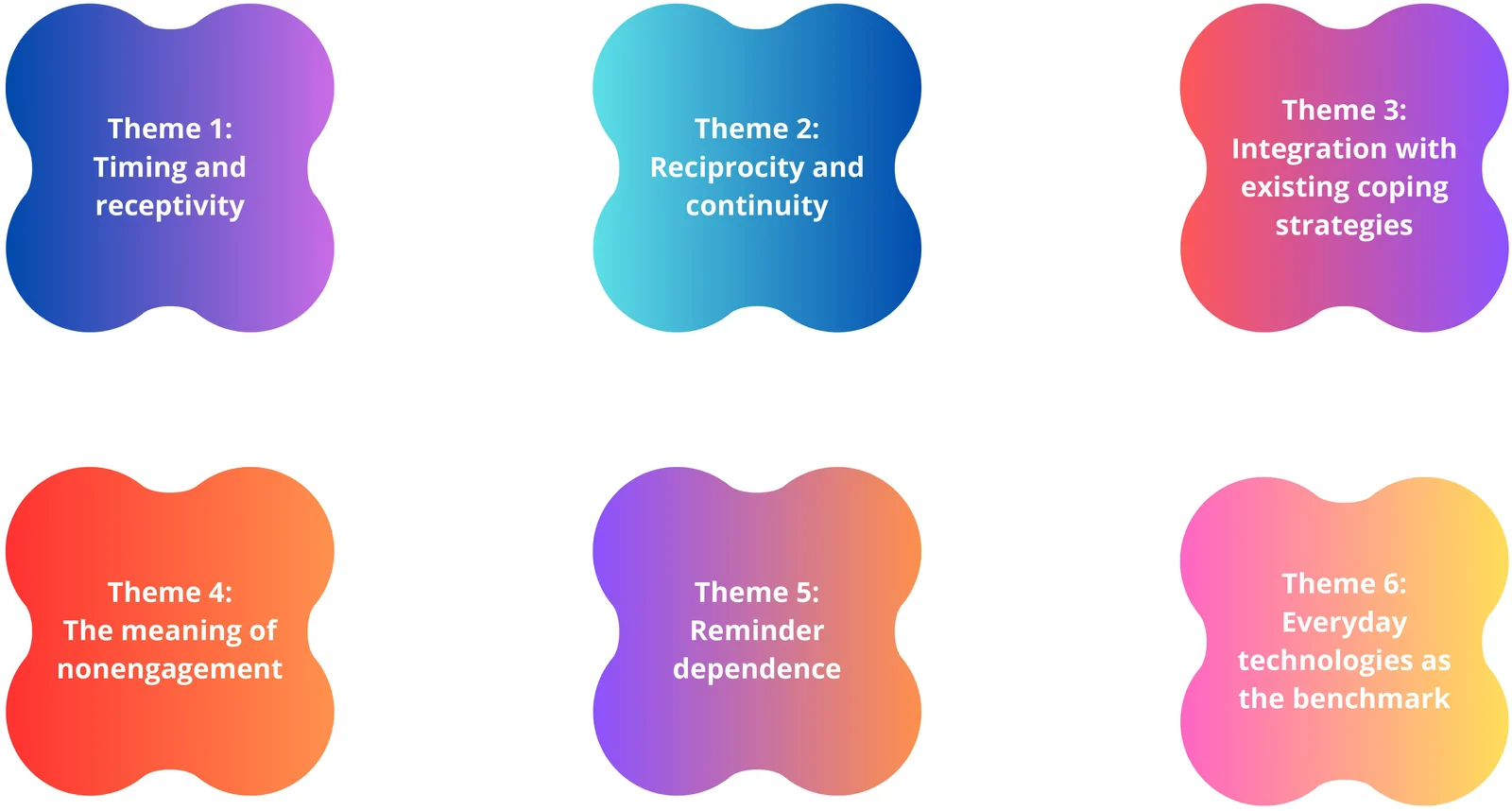
Overview of the 6 themes identified through collaborative reflexive thematic analysis (RTA).

### Theme 1: Timing and Receptivity

From the first few days, the timing of MINT’s notifications made a noticeable difference in how we received its exercises. There were moments when an intervention arrived just as we were settling into routines we had intentionally carved out for ourselves, like cooking dinner or finally sitting down to relax. RP2 captured this most vividly: “Very often [notifications from MINT] arrived when I was doing something else… For example, one day I had just turned on the Xbox, I was finally relaxing, and the reminder came. At that moment the bot was stealing the only space of peace I had managed to carve out.” That feeling of intrusion was shared by all of us: even brief prompts could turn a low-stress moment into a jolt of irritation, not relief. But it was not only the schedule that mattered; our emotional state at the moment of delivery was equally decisive. Exercises framed with positivity, like visualizing the best possible self or the smiling exercise, were polarizing. When we were relatively calm, these exercises could feel energizing or pleasant. But on days when tension was high, the same prompts sometimes backfired. RP4 put it perfectly: “The best possible self is a perfect example of a double-edged tool… when I was already feeling down it made me feel worse, because I was imagining a version of myself that was completely unreachable at that moment.” Smiling exercises could feel hollow or even irritating under stress, while other exercises, like controlled breathing, felt neutral and safe, something we could do without pretending that everything was fine. They demanded little emotional effort and could be accessed regardless of mood. These experiences showed us how timing and emotional alignment worked together as gatekeepers. The same exercise could either be welcomed or rejected depending on when it arrived and how we felt. For instance, even an exercise we normally enjoy could trigger annoyance if it appeared at a moment when our attention or emotional bandwidth was low. As the weeks went on, we noticed this pattern more clearly. We started anticipating how we might feel before even opening MINT, sometimes ignoring notifications in advance, preemptively judging the exercise’s fit with our current state. Over time, we realized that acceptability was not just about content, but about context, about how each exercise intersected with the unpredictable rhythms of daily life.

### Theme 2: Reciprocity and Continuity

Another recurring experience concerned the balance between effort and return. Using MINT required regular attention: daily EMAs, diary entries, exercise completion, and occasionally syncing wearables. Yet, despite investing this energy, there was little sense that the bot responded to us or incorporated our input in any meaningful way. RP5’s reflection expressed this plainly: “You give the chatbot a lot of data but you get nothing back… no real insight.” This sentiment resonated across the group. At times, the bot felt like an “empty box” (RP2) or “just another sequence of homework,” (RP5), with no acknowledgment of what we had already done. Over time, we realized how much relational cues mattered. Small conversational touches, such as brief comments, acknowledgment of previous entries, and even light humor, could have made exercises feel less transactional. RP2 captured this perfectly: “The role of small talk is fundamental… Those circumstantial chats are what unblock everything in in-person counselling sessions. If you arrive at the therapy room and the counselor immediately applies a cognitive-behavioural technique, you feel you are only receiving directives.” We noticed that without any sense that the bot “knew” us or remembered our past engagement, its exercises sometimes felt cold, prescriptive, and burdensome. Some of us also wanted visible feedback as evidence that our engagement mattered. RP1 wrote in her notes, “I would have loved a simple dashboard, even just a weekly message saying ‘Last week you felt better on the days you walked more … shall we use that?’… something that shows the bot is actually doing something with everything I’m giving it.” Without this, even well-designed exercises sometimes felt like chores. Over time, this shaped how and when we engaged, as the perceived effort of participation without visible reciprocity began to weigh more heavily than the potential benefits of the exercises themselves.

### Theme 3: Integration With Existing Coping Strategies

The moments when we truly felt the tension dissolve rarely lined up with finishing a MINT exercise. More often, relief arrived through small, familiar things we reached for on our own, like going for a run (RP1), walking the dog in the evening light (RP3), turning the car into a private concert hall and singing at the top of our lungs while stuck in traffic (RP2), curling up with a novel for 10 quiet pages (RP4), sketching, or simply loading a favorite video game. These strategies also shared another characteristic: they worked quickly and intuitively. RP1 noted, “I already know what works for me … running gives me the endorphins, loud rhythmic music settles my breathing, a few pages of fiction melt the noise in my head… it just does.” Even when MINT exercises were tailored to our VARK preferences, they often felt slower or more effortful compared with strategies we had already internalized. We noticed ourselves comparing the 2: the structured exercises vs our own spontaneous coping tactics. The comparison was stark: while the exercises were often interesting or novel, they did not always provide the immediate, visceral relief that our own strategies did. Over time, we started to integrate MINT exercises selectively, favoring moments when our usual strategies were unavailable or when we wanted to experiment with a different approach. This realization prompted deeper reflection on how we individually manage stress and how digital interventions fit into those practices. It became clear that while MINT offered structured, evidence-based exercises, the most meaningful support often came from us and the routines we had developed over years of trial and error. Over the weeks, our engagement with the bot shifted from following instructions to observing, reflecting, and occasionally experimenting, blending our spontaneous coping strategies with its prompts when it seemed useful.

### Theme 4: The Meaning of Nonengagement

One pattern became impossible to ignore over the 4 weeks. Our engagement, or lack of it, was closely tied to moments of stress. The periods when we did not interact with MINT often turned out to be the most informative. On low-stress days, we were conscientious, completing EMAs, logging diary entries, and experimenting with exercises. Yet, when stress peaked, our interactions dropped sharply. RP2 described this clearly: “My engagement pattern made me think of missing not at random [MNAR] … the classic example to understand MNAR is the alcoholic who doesn’t fill in the questionnaire when he drinks. Here, when I was really overwhelmed, I simply didn’t record anything.” This was true for all of us: periods of silence were rarely accidental; they were protective withdrawals from additional effort or exposure to stress. For instance, one of us (RP3) missed every EMA on the day of a major work crisis, while another (RP2) did not engage with interventions and left diary entries blank for 3 consecutive days, later recalling them as “the worst [part] of the entire month.” In these moments, engaging with MINT felt like adding friction rather than offering relief. As we revisited our diaries, EMAs, and workshop discussions, the blank spaces themselves became meaningful signals. They came to represent moments when the support we most needed was, paradoxically, the hardest to access. We began to treat missing entries as informative, signaling not a failure of adherence but the contours of our emotional landscape. Interestingly, these gaps also shaped how we thought about the system’s design. We realized that MINT had no way to detect absence or adapt when we withdrew. In contrast to times when engagement was high, peak stress moments were invisible to the system, and its push notifications often went unanswered, further emphasizing the mismatch between system design and lived reality. By the end of the study, we collectively understood that these silences were not just “missing data” in a technical sense; they were data in themselves, communicating both our states of mind and our coping capacity. Recognizing this made us reflect differently on the metrics of success for digital interventions: completion rates alone were insufficient. The pattern of nonengagement became a story in itself, narrating where MINT could not reach us and where the system might have needed to adapt.

### Theme 5: Reminder Dependence

Week 4 was revealing for us in a way we hadn’t anticipated. We made a provocative and deliberate choice: at the start of the week we switched off every scheduled notification overnight. No gentle tapering, no transitional phase, just silence. In real-world deployments, this would be unusual and potentially harmful, but for us, it was intentional. We wanted to force the question: once the external scaffolding disappears, what remains? The answer came within hours and could not have been clearer.

Within 2 days, 4 of the 5 participants had essentially abandoned the bot (Figure 3). As RP2 summed it up in the second workshop, “Without reminders the intervention crashed… we all crashed, except you RP4.” RP4 was the quiet exception. She continued to use controlled breathing and deliberate walking at genuine moments of tension. The techniques had become hers; the bot had successfully faded away. For the rest of us, when the prompts vanished, so did any impulse to seek the bot out. The radical week 4 switch acted like a stress test that exposed a truth we would otherwise have discovered only after months of gradual disengagement: the first 3 weeks had trained dependence, not independence. We had inadvertently built a system that functioned like a daily pill, something you take because it arrives, not because you have learned to live differently. Curiosity and our duty as researchers had kept us compliant, but MINT had never become a companion in genuine behavior change. This realization hit hard. Microintervention systems are often conceived as short-term analgesics: deliver evidence-based techniques, reduce symptoms, repeat. But week 4 showed that symptom relief without skill transfer is little more than digital pharmacology: effective only as long as the external dose keeps coming. True behavior change companions, by contrast, must do the opposite of a pill: they must work themselves out of a job by helping the person internalize new patterns until the tool itself becomes optional. RP2 articulated how we should have used those crucial early days instead: “The week should be a protected onboarding and alliance-building phase. The bot should be deliberately present, even chatty, but with one clear message: ‘For the next week I’ll be around a lot, not to give you homework, but so we get to know each other and so you learn exactly how to use me when you actually need me.’ It teaches transparently: how stress works in the body, why breathing is an instant reset, why movement is medicine. It gives three or four one-word commands (‘BREATHE,’ ‘WALK,’ ‘PAUSE,’ ‘VENT’). Every evening it asks one gentle question: ‘One thing that helped today?’ or ‘Anything you want me to remember?’ By the end of that week the user trusts the bot, feels seen, and knows precisely how to call for help. Only then do you start fading the prompts.” The abrupt week 4 silence was therefore not a flaw; it was the perfect diagnostic. By removing the external “dose” all at once, it revealed that we had built a pharmacological tool rather than a behavior change companion. It proved that genuine autonomy is cultivated from day 1 through transparency, psychoeducation, felt understanding, and explicit pathways for self-initiation. In other words, our prototype taught us how to design another version of it that, like the best therapist or coach, eventually makes itself proudly obsolete.

**Figure 3.**
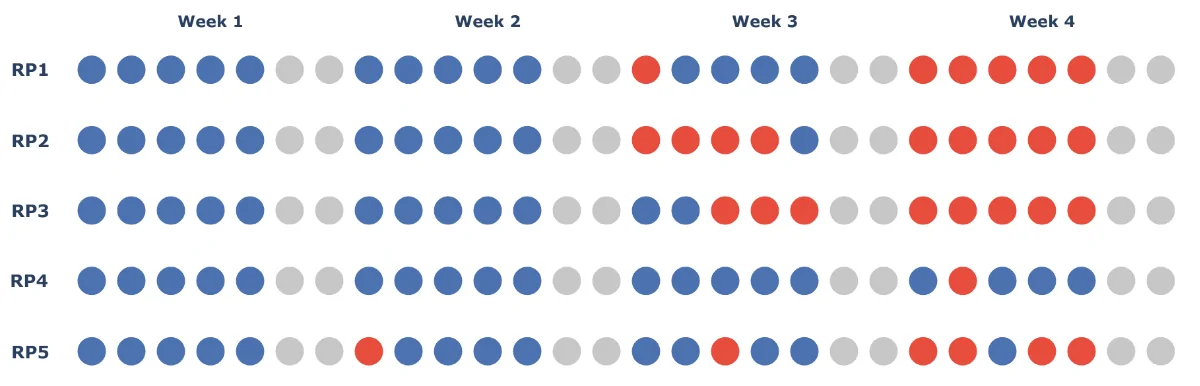
Participant timeline showing daily engagement patterns (completed microinterventions) with the MINT (Micro-Interventions for Navigating Tension) chatbot. Red indicates that the RP did not engage with MINT, whereas blue indicates that the RP engaged with MINT. RP: researcher-participant.

### Theme 6: Everyday Technologies as the Benchmark

Finally, 3 of us (RP2, RP3, and RP4) wore wearable devices to capture physiological signals during the study. In practice, the data provided by those devices proved far less useful than we had hoped. Signals were noisy and frequently mismatched with how we actually felt. As RP2 recalled a frightening moment on the road, “A car almost hit me on a bend… I expected a massive peak [in EDA]… instead I saw nothing at all.” RP4 regularly experienced the reverse: “I felt calm and it said I was super stressed…” Practical difficulties added to the disappointment. Two of us (RP1 and RP4) developed skin irritation within the first week, even with the medical-grade device, and daily charging became another small but persistent hassle. By the second workshop, we agreed that, for the moment, wearable-derived stress metrics remain too inconsistent and burdensome to serve as a reliable foundation for everyday intervention. They generated interesting patterns for post hoc reflection but could not be trusted in real time. Wearables, however, were not the only benchmarks that shaped how we experienced MINT. An even more unexpected comparison emerged from our own everyday habits. We ourselves, while testing MINT, began turning to general-purpose LLMs for the emotional support that the prototype was not providing. As RP4 admitted with a half-smile, “I write everything I’m feeling on ChatGPT… it helps just to put the words down… it’s my free psychologist … well, cheap psychology anyway. [laugh]” The joke landed because it was true for several of us (RP2, RP3, and RP4). During the same 4 weeks we were dutifully completing MINT’s EMAs and exercises, we were also quietly opening ChatGPT (OpenAI) to vent after a hard day, draft angry messages more calmly, externalize racing thoughts, or simply to feel heard when the structured bot stayed silent. These parallel conversations were never part of the protocol; they happened spontaneously, often late at night or in moments of peak frustration. Some exchanges were brief check-ins, others long, meandering threads that continued over days. We noticed the same patterns: the model remembered what we had said before, asked gentle follow-up questions, and responded with a warmth that, however simulated, felt caring. A few of us also observed that, at times, chatting with an LLM could have downsides, like enabling prolonged rumination rather than gently interrupting it. Instead of recognizing the loop and helping the person close or rework the situation, the model simply kept the conversation going. We had deliberately designed MINT to be short, focused, and emotionally neutral in order to respect time and clinical rigor. In practice, we found ourselves seeking out something more verbose, curious, and unmistakably human-like. Current openly available LLMs, tools never intended for therapeutic use, quietly showed us the level of continuity, attentiveness, and conversational space that users now expect from any digital companion. The unreliable wearables and our own unsolicited use of LLMs reminded us that users (even when the users are the designers) do not evaluate a new intervention in isolation. We compare it, consciously or not, with everything else already in our hands.

## Discussion

### Principal Results and Comparison With Prior Work

#### Contribution of CAE

Digital mental health research has already identified many of the challenges discussed in this paper, including issues related to timing, engagement, personalization, attrition, and the limitations of current sensing technologies. We do not present these themes as entirely new findings. Their value lies instead in the level of detail with which CAE allowed us to examine them from the inside: not only as design problems but also as experiences that unfolded, accumulated, and changed meaning across ordinary days. As anticipated in the *Introduction*, traditional trials, log analyses, and feasibility studies can show when engagement drops, which prompts are completed, and which features are preferred. CAE allowed us to ask a different question: what does it feel like when those patterns happen, and how do users make sense of them while they are living with the system? These forms of evidence are complementary rather than competing. In this discussion, we therefore use the literature as a point of orientation but focus on what the CAE perspective added to each theme: the mechanisms, tensions, and lived interpretations that are often flattened in aggregate engagement metrics.

#### Theme 1

The first example is about timing. JITAI research has already shown that receptivity depends strongly on when support is delivered [36]. Poorly timed prompts can reduce engagement, while better-timed ones can make digital support feel more relevant. What our CAE made visible, however, was the deeper meaning of “poor timing.” A mistimed prompt was not simply inconvenient. At times, it interrupted a moment participants had actively created to recover from stress. This matters because the system was not only competing with work, distraction, or busyness; it was also sometimes competing with users’ own successful attempts at restoration.

This also aligns with the occupational psychology literature on the importance of psychological detachment and psychological restoration, which underscores the importance of preserving personal downtime for the recovery of depleted cognitive and emotional resources [37,38]. From this perspective, the design issue is not just interruption management; it is the risk that an intervention aimed at supporting regulation may disturb an ongoing act of self-regulation.

The CAE also helped us distinguish need from receptivity. In everyday use, higher stress did not automatically translate into greater openness to support. Some exercises required a degree of emotional availability that was absent precisely when stress was high. Others, especially lower-effort regulation strategies such as breathing, felt easier to access across a wider range of states. This points to a narrow and unstable window: enough distress for support to matter, but enough attentional and emotional bandwidth to engage with it. Current wearable approaches often equate physiological arousal with need [39-41], but our experience suggests an inverted-U pattern: too little arousal yields little or no benefit, whereas excessive arousal triggers protective withdrawal; this underscores why linear “more arousal equals more need” algorithms risk delivering interventions precisely when users are least able or willing to receive them.

#### Theme 2

Findings on the perceived lack of reciprocity and relational cues instead extend previous work showing that relational agents support sustained engagement [42,43]. Our experience suggests that the issue goes beyond conversational style. Throughout the 4 weeks, participants rarely questioned the quality of the exercises themselves. Instead, frustration accumulated because the effort invested in the interaction seemed to disappear into the system. Logging moods, completing EMAs, and recording reflections gradually came to feel like one-way transactions, with little indication that MINT was doing anything with that information. Seen in this light, reciprocity is not simply about making a chatbot warmer or more socially engaging. It is about making users feel that their contributions have consequences. Even small signals (eg, a reference to a previous interaction, a brief acknowledgment, or a simple synthesis of recurring patterns) could have changed how participants interpreted the same interaction. The absence of these cues shifted the perceived balance between effort and value, until the exercises themselves began to feel increasingly transactional. This resonates with previous work showing that users disengage when conversational agents fail to establish a sense of relationship or continuity [44,45]. Reading back the workshops, what stood out was that we rarely asked for a more empathic chatbot. We asked for signs that the chatbot was listening. Those are related ideas, but they are not the same. The issue was less the absence of warmth than the absence of evidence that previous interactions had shaped subsequent ones.

#### Theme 3

Theme 3, on the other hand, resonates with longitudinal studies showing that people rarely adopt the full repertoire of techniques offered by mindfulness or CBT apps, instead selecting only those that fit their everyday lives [46,47]. Our experience suggests that this selectivity is not simply a matter of personal preference. Throughout the study, participants seemed to evaluate each proposed exercise against an already existing repertoire of coping strategies that had been built through years of experience. The decision was rarely “Is this exercise good?” It was closer to “Is this more useful than what I would naturally do right now?” Familiar activities such as walking, running, reading, or listening to music were not necessarily perceived as more evidence-based or therapeutically sophisticated. They simply required little deliberation, carried no learning cost, and already belonged to participants’ habits. Structured microinterventions, therefore, entered an environment where effective coping strategies were already in place, rather than an empty space waiting to be filled. This perspective is consistent with the person-based approach to intervention development [48], which emphasizes understanding how interventions fit within people’s existing lives rather than expecting people to adapt to the intervention. Our findings reinforce this principle by suggesting that future systems should explicitly discover and build upon users’ own coping repertoires from the outset. This does not reduce the value of evidence-based exercises. Instead, it positions them as additions to an existing repertoire, introduced when they genuinely expand what users can already do rather than asking them to replace or take the place of strategies that already work.

#### Theme 4

Nonengagement during periods of elevated stress is a phenomenon that has already been described as a form of MNAR data in digital mental health research literature [49], and similar patterns have been reported in studies of depression and bipolar disorder, where drop out often coincides with symptom worsening [50,51]. Even in this case, our experience did not challenge this interpretation. It added something more specific: the opportunity to understand what those missing moments actually represented from the participant’s perspective. Looking back together at diaries, workshop discussions, and EMA records, we found that periods of nonengagement rarely appeared as simple lapses in adherence. They reflected moments in which interacting with the system itself had become one demand too many. This perspective echoes recent calls to treat nonuse as meaningful data rather than simply as adherence failure [49,52,53]. Our findings suggest that the next step is not only to detect these patterns computationally but also to understand the circumstances that produce them. From a design perspective, prolonged silence should perhaps be interpreted less as disengagement from the intervention itself and more as a potential indicator that the demands of engaging with the intervention have temporarily exceeded the user’s available capacity.

#### Theme 5

Similarly, the sharp decline in self-initiated use after scheduled reminders were removed reflected a challenge that is increasingly being documented across many DMHIs. Longitudinal evaluations of commercial apps consistently report high initial engagement followed by substantial attrition over time [54,55]. Our experience suggests that this transition deserves attention not only as an engagement problem but also as a question of learning (in the sense of internalizing intervention strategies). What remains once the external structure disappears? Considering this theme alongside theme 3 offers 1 possible interpretation. Throughout the study, participants continued to rely on coping strategies that had become part of their everyday routines through years of personal experience. By contrast, most of the chatbot exercises remained closely associated with the chatbot itself. They were completed when prompted, but they rarely became something participants reached for independently. RP4 was the only participant who continued using exercises after the reminders had disappeared. In her case, the strategies had become detached from the chatbot that had introduced them and had entered her everyday coping repertoire. This distinction may be more important than continued use of the chatbot itself. A successful intervention is not necessarily one that people keep opening indefinitely but one whose underlying strategy gradually becomes part of their own repertoire. When that transfer does not occur, engagement risks remaining dependent on the external structure that originally sustained it.

#### Theme 6

Finally, theme 6 broadens the lens beyond the chatbot itself. As we reflected on our experience, it became increasingly clear that we were not evaluating MINT against an abstract idea of a digital intervention. We were comparing it, often implicitly, with technologies that had already become part of everyday life and had quietly reshaped our expectations. General-purpose LLMs were the clearest example of this shift. We did not expect MINT to become an open-ended conversational agent, nor did we see that as its purpose. Instead, interacting daily with LLMs had changed what a conversational system was expected to do. When read alongside theme 2, the issue was not simply conversational warmth. It was continuity. We expected signs that previous interactions still mattered: that the chatbot remembered, connected, and built on what had already been shared. As these behaviors become commonplace in everyday AI systems, their absence becomes more noticeable even in highly structured digital health interventions. Recent work is beginning to document similar shifts in users’ expectations of specialized mental health tools [56-58].

Just as LLMs have changed our expectations of conversation, the increasing presence of wearable devices in our lives has also shaped our expectations of physiological sensing. Simply wearing a device that continuously monitors physiological signals creates the expectation that the system will recognize meaningful changes in one’s state. Across our reflections, the critical issue was not occasional inaccuracies themselves but what happens after repeated mismatches between sensed data and lived experience. Those moments gradually weaken confidence that the system can recognize when support was actually needed. Previous work has extensively discussed the technical challenges of using consumer wearables for stress detection in everyday settings [59-61]. Our experience suggests an additional consequence that is less often examined: repeated mismatches may gradually erode users’ willingness to rely on physiological sensing as the basis for intervention delivery. From the user’s perspective, sensing is not evaluated signal by signal but through the accumulation of moments in which physiological measures either correspond or fail to correspond with lived experience.

### Design Implications and Directions for Future Research

Designing DMHIs often begins with deciding what support to provide. Our experience suggests that just as much attention should be given to what happens after that support is introduced into everyday life. Many of the design implications that emerged from this study concerned not the content of the interventions themselves but the conditions that shaped how they were experienced and, over time, whether they became part of everyday routines. To translate these observations into practical guidance for both the further development of MINT and the design of future digital stress-management interventions, Table 3 synthesizes the key design challenges emerging from our findings together with their corresponding design implications.

**Table 3.** Design implications for digital microintervention systems for stress management derived from the 6 collaborative autoethnography (CAE) themes.

Design focus	Key empirical insight	Design implication
Receptivity-aware intervention delivery	Theme 1	Develop receptivity-aware triggering that goes beyond physiological arousal, estimating not only when support is needed but also when users are likely to have sufficient emotional and attentional capacity to engage with it.
Relational continuity and reciprocity	Theme 2	Treat continuity across interactions (eg, remembering previous inputs and acknowledging earlier exchanges) as a core design feature rather than a conversational enhancement.
Integration with existing coping strategies	Theme 3	Design microinterventions to extend users’ existing coping repertoires rather than compete with or replace them.
Accounting for nonengagement	Theme 4	Redefine nonengagement patterns as meaningful signals (MNAR^a^) that should inform adaptation and evaluation, rather than interpreting them only as adherence failure.
Transition to autonomous use	Theme 5	Design explicit pathways through which users gradually move from prompted engagement to self-initiated coping, allowing reminders to fade as strategies become part of everyday life.
Appropriate use of wearable sensing	Theme 6	Use wearable sensing primarily to enrich contextual understanding rather than as the sole trigger for intervention delivery in everyday settings.
Balancing everyday AI expectations with evidence-based interventions	Theme 6	Recognize that users increasingly expect continuity across interactions and explore architectures that can provide it while remaining clinically appropriate.

a MNAR: missing not at random.

Several questions naturally follow from these observations. One concerns receptivity: future work should explore how multimodal sensing and lightweight contextual information can better estimate when support is both needed and welcome. Another concerns learning over time. Combining microrandomized trials with longer qualitative follow-up could help clarify how people move from externally prompted engagement to genuinely self-initiated use. Finally, the widespread adoption of everyday generative AI raises new design questions. Hybrid scripted-generative architectures [62,63] offer one promising direction for exploring how conversational continuity can be achieved while preserving the structure and safety of evidence-based interventions.

### Limitations and Role of CAE in Digital Health Research

Before concluding, it is important to acknowledge several limitations of this study. First, our RPs were all digital mental health researchers, and this inevitably shaped both the usage of the prototype and the depth of interpretations. Compared with the broader population, we were likely more attentive to design decisions, more accustomed to reflecting on our own engagement, and more familiar with stress-management strategies. The study also lacked, by definition, cultural, socioeconomic, and clinical diversity, and RP2’s dual role as a prototype developer may have influenced parts of the collective sense-making despite our explicit attention to positionality throughout the study. The research lasted 4 weeks, longer than most feasibility pilots but still short relative to the development of longer-term habits. Importantly, because our analysis of nonengagement (theme 4) relies on retrospective self-reports and collaborative reflections, the study design cannot conclusively determine the causes of nonuse or distinguish whether missed interactions were driven by emotional overload or by competing daily demands, participant burden, and reduced motivation over time. These observations should therefore be understood as subjective participant interpretations rather than empirically established causal claims. In addition, MINT relied on scheduled rather than stress-contingent intervention delivery. This was an intentional design choice, as our goal was to understand how scheduled microinterventions are experienced in everyday life rather than to evaluate JITAI algorithms. Likewise, the abrupt removal of reminders during the final week was designed to probe self-initiated engagement and whether participants had integrated the strategies into their everyday coping repertoire, not to simulate a realistic deployment strategy. As a result, the observed decline in engagement cannot be attributed solely to reminder dependence but should instead be interpreted as highlighting the need to better understand how users transition from externally prompted support to independent use, including within more context-aware intervention designs. At the same time, this study also illustrates what CAE can contribute during the early stages of designing digital health interventions. Several of the insights discussed in this paper did not emerge from single interactions but from returning to the same experiences over time and discovering that they acquired a different meaning once considered alongside later events. What initially felt like isolated moments of frustration, comfort, or disengagement gradually became interpretable as recurring patterns. This depth of interpretation depended not only on prolonged engagement with the system but also on the collective sense-making that sits at the heart of CAE. Revisiting experiences together allowed us to question initial interpretations, surface alternative readings, and gradually build richer explanations than any one perspective could have produced alone. Perhaps the most valuable aspect of this process was not that it generated more data but that it gave us repeated opportunities to elaborate on what those data meant. For us, this reinforces the role of CAE as a complementary approach within the broader evaluation of digital health interventions, particularly during the early stages of design, where understanding how design decisions are experienced can meaningfully inform what is tested next.

### Conclusions

This study used CAE to examine the everyday experience of using a stress-management chatbot over 4 weeks. Using MINT as part of our daily routines allowed us to look beyond individual microinterventions and consider how they were experienced over time: how expectations developed, how support was interpreted in different situations, and how the strategies introduced by the chatbot related to our existing ways of coping. The study also illustrates what CAE can contribute to the evaluation of digital health interventions. Returning to the same experiences over time and making sense of them collectively often enriched our initial interpretations. Experiences that initially seemed unrelated acquired a different meaning when revisited alongside later events, revealing patterns that were difficult to recognize in the moment. Instead of introducing entirely new design challenges, this process helped us develop richer interpretations of familiar ones, moving beyond description toward explanations and hypotheses that can inform subsequent stages of intervention design and evaluation. We see this as complementing more established evaluation approaches, particularly during the formative stages of design. More broadly, this experience reminded us that design features rarely have a single, fixed meaning. The same feature can be reassuring, frustrating, motivating, or simply irrelevant depending on when it is encountered, what has happened before, and how it fits within the broader context of everyday life.

## Supplementary material

10.2196/89876 Multimedia Appendix 1 MINT (Micro-Interventions for Navigating Tension) study implementation files.

10.2196/89876 Multimedia Appendix 2 Reflexive thematic analysis outputs.

## Abbreviations

BFI-10: Big Five Inventory-10
CAE: collaborative autoethnography
CBT: cognitive behavioral therapy
COPE-28: Coping Orientation to Problems Experienced-28
DMHI: digital mental health intervention
EDA: electrodermal activity
EMA: Ecological Momentary Assessment
EMI: ecological momentary intervention
HRV: heart rate variability
JITAI: just-in-time adaptive intervention
LLM: large language model
MINT: Micro-Interventions for Navigating Tension
MNAR: missing not at random
REIm-13: Rational Experiential Inventory-13
RP: researcher-participant
RTA: reflexive thematic analysis
VARK: visual, aural, read/write, kinesthetic
