# Supplementary material for: Insights on Designing the MINT (Micro-Interventions for Navigating Tension) Chatbot System for Stress Management: Collaborative Autoethnography
Source: JMIR Hum Factors. 2026 Oct 27;13:e89876. doi: 10.2196/89876 (PMC13717341; doi:10.2196/89876)
Supplement: Supplementary file 1 [file humanfactors-v13-e89876-s001.docx]

**Multimedia Appendix 1. MINT Study Implementation Files**

This multimedia appendix provides the machine-readable materials used to implement the MINT study, including the intervention logic, ecological momentary assessment (EMA) flows, and study schedule. The files are shared to support transparency, reproducibility, and reuse.

Contents:

- MINT Intervention Flows (JSON)
- MINT EMA Flows (JSON)
- MINT Schedule (CSV)

All files are available at: <https://osf.io/c9gha>
