# Supplementary material for: Insights on Designing the MINT (Micro-Interventions for Navigating Tension) Chatbot System for Stress Management: Collaborative Autoethnography
Source: JMIR Hum Factors. 2026 Oct 27;13:e89876. doi: 10.2196/89876 (PMC13717341; doi:10.2196/89876)
Supplement: Supplementary file 2 [file humanfactors-v13-e89876-s002.docx]

**Multimedia Appendix 2. Reflexive Thematic Analysis Outputs**

This multimedia appendix presents the results of the reflexive thematic analysis conducted on researcher-participant data. It includes the identified themes and subthemes, illustrative quotes, and the distribution of codes across the dataset to support analytic transparency.

Table MA2-1. Themes, subthemes, illustrative quotes, and coding distribution identified through reflexive thematic analysis of researcher-participant data.

| **Theme** | **Sub-theme** | **Evidence Verbatim** | **# Distinct coding instances** | **# RP voicing this sub-theme** |
| --- | --- | --- | --- | --- |
| T1. Timing and receptivity | ST1.1. Interruption of self-carved recovery moments | *“The intervention sometimes arrives while you’re doing something else. For example when you’re at the supermarket and can’t do it, or you’re already doing something relaxing like reading a book” - RP1*  *“It interrupted moments when I had finally carved out some calm” - RP4* | 8 | 4 |
|  | ST1.2. Emotional incongruence with current mood | *“The same exercise can feel completely different: positive when I’m already okay, but when I’m really stressed it actually makes things worse” - RP5*  *“When there’s too big a gap between how I feel and what the exercise is asking me to do, I just can’t get into it” - RP3*  *“If I was having a worse day, an exercise I liked the day before suddenly felt forced” - RP4* | 6 | 4 |
|  | ST1.3. Inverted-U receptivity window | *“If you’re not stressed at all it feels pointless; if you’re way too stressed you don’t have the energy to do it” - RP2*  *“When the stress is sky-high you simply don’t do the exercise” - RP1* | 4 | 3 |
| T2. Reciprocity and continuity | ST2.1. Talking into a void | *“The data just stay there, they don’t come back to you in any useful way” - RP1*  *“I didn’t feel understood or connected at all” - RP5* | 4 | 3 |
|  | ST2.2. Missing relational cues and small talk | *“This chatbot completely skipped the small-talk phase that builds rapport” - RP2*  *“It felt more like a homework assignment than a companion” - RP5*  *“There was no therapeutic alliance with the bot” - RP1* | 4 | 3 |
|  | ST2.3. Desire for visible feedback from own data | *“It would be useful if the insights from my mood tracking came back to me as a dashboard” - RP1*  *“If you don’t get any summary or reflection on the data you entered, there’s no incentive to keep doing it” - RP5*  *“After a few EMAs it could just say ‘this week was better/worse than last week’” - RP2* | 4 | 3 |
| T3. Integration with existing coping strategies | ST3.1. Spontaneous strategies provide faster/more intuitive relief | *“Listening to the radio, playing video games, walking the dog … those are the things that actually help me instantly” - RP1*  *“I automatically started walking when I felt very stressed” - RP4*  *“I turned the radio up loud in the car and suddenly the traffic jam felt enjoyable” - RP1* | 6 | 3 |
|  | ST3.2. Structured exercises feel more effortful or forced | *“Some exercises, even if I normally like them, felt forced or unnatural depending on my mood that day” - RP4* | 4 | 3 |
| T4. The meaning of non-engagement | ST4.1. Protective withdrawal during peak stress | *“When I was really stressed I didn’t want to think about it at all, so I just didn’t log anything” - RP4*  *“Asking me to rate my stress or do an exercise when I was already at breaking point felt like one more demand I couldn’t meet.” - RP2* | 4 | 3 |
|  | ST4.2. Silences as MNAR signals | *“Today is gonna be a MNAR as I have piled up too much stress and I just want to disconnect” - RP2*  *“I didn’t log the really bad moments because thinking about it would make it worse.” - RP4* | 4 | 2 |
| T5. Reminder dependence | ST5.1. Rapid collapse when reminders were removed | *“Without reminders the week just flies by and you realise you haven’t done anything” - RP1* | 5 | 5 |
|  | ST5.2. Reminder dependence rather than skill internalisation | *“We spent three weeks training ourselves to wait for a ping instead of building an internal trigger. No wonder everything collapsed when the pings stopped.” - RP2*  *“We accidentally designed digital pharmacology instead of a coach that teaches independence” - RP1* | 4 | 3 |
|  | ST5.3. Optimization of early days | *“It would have been much better to have an initial phase where the bot learns what already works for me (walking, music, gaming…) and then uses those.” - RP4*  *“I think 2 weeks would be the maximum amount of time to deliver educational content. After two weeks I see nothing more than a pull system…” - RP2* | 4 | 3 |
| T6. Everyday technologies as the benchmark | ST6.1. Consumer wearables currently unreliable and burdensome | *“I got a rash from wearing it” - RP2*  *“The data were completely random: calm when I was stressed and vice versa” - RP4*  *“Even a near-accident didn’t register any physiological spike” RP2* | 7 | 2 |
|  | ST6.2. Spontaneous turn to general-purpose LLMs for relational support | *“I write how I’m feeling to ChatGPT … it says ‘I understand you’, and that alone helps” - RP4*  *“People are already using it as a cheap therapist” - RP1*  *“I use it to check my angry messages before sending, it triggers useful reflection” - RP2* | 5 | 3 |
